# Weight Loss and Mortality

**DOI:** 10.1371/journal.pmed.0020200

**Published:** 2005-06-28

If you are overweight, then losing weight is good for your health, surely? Unfortunately, the evidence on which an answer to this seemingly simple question might be based is at best equivocal, and at worst very controversial. Previous work has shown that weight loss in obese people improves risk factors associated with cardiovascular diseases and diabetes, but studies are conflicting on the long-term effects of weight loss on mortality. A study in this month's *PLoS Medicine* by Jaakko Kaprio and colleagues on a Finnish dataset adds more evidence to this debate, but experts are divided on what can be concluded from it.

The major difficulty in getting clear results on this question is that it is virtually impossible to do a controlled trial to answer it. Hence, the evidence accumulated has come mostly from epidemiological studies, but it is notoriously difficult to remove all confounding factors from these studies. Kaprio and colleagues' study is another epidemiological study, but we should not simply dismiss the data as unreliable just because of the problems inherent to such a study design. Instead, we should consider their study in the light of all the other evidence available.

Starting from a group of 19,993 twins from Finland who have been studied since 1975, the authors gathered data from the 2,957 overweight participants who remained after they had excluded people with pre-existing disease, and those with missing data. These twins had been asked in 1975 if they intended to lose weight, and then had information on weight collected in 1981. Information on mortality was then collected over the next 18 years; the authors then analyzed mortality in relation to intention to lose weight and actual weight change.

In total, 268 people died. When the results were analyzed, the surprising finding was that people who intended to lose weight, and who did so, had a somewhat higher mortality than those who intended to lose weight but whose weight remained stable, or went up. People who intended to lose weight, and who did so, also had a slightly higher mortality than those who did not intend to lose weight and whose weight was stable.

The problems with such a study are outlined in an accompanying Perspective (DOI: 10.1371/journal.pmed.002018) by Meir Stampfer from Harvard School of Public Health, and there is no doubt that these results seem counterintuitive. Some readers may take away the idea from this paper that overweight people should not be advised to lose weight, but Stampfer cautions against that interpretation. Perhaps the safest interpretation of these results is that by the time adults are overweight, the health benefits of losing weight are not clear-cut. If there is one message therefore that should be taken from the paper it is this: in order to prevent the associated health effects of obesity, preventing obesity, especially in childhood, should be an overriding public health priority.

The study leaves us with the question of how intentional weight loss could lead to excess mortality. The authors suggest that this could be due to the unavoidable loss of lean body mass, which according to several other studies may increase mortality, and which may outweigh the beneficial effects of losing fat mass in healthy individuals. The authors therefore conclude that “the long-term effects of weight loss are complex, and they may be composed of oppositely operating effects with net results reflecting the balance between these effects.”

